# Supplementary material for: Crowding-induced transcriptional bursts dictate polymerase and nucleosome density profiles along genes
Source: Nucleic Acids Res. 2017 Jun 6;45(13):7623–32. doi: 10.1093/nar/gkx513 (PMC5737439; doi:10.1093/nar/gkx513)
Supplement: Supplementary Data [file gkx513_supp.pdf]

# Supplementary material

## Crowding-induced transcriptional bursts dictate polymerase and nucleocome density profiles along genes

Aafke A van den Berg and Martin Depken

We here give the derivations and arguments that were left out of the main text for clarity. For brevity we will here only keep the 'b' of 'bulk' in the subscript, for example writing $v^{\mathrm{b}}$ instead of $v^{\mathrm{bulk}}$.

## 1 Heuristic solution for of the hierarchical TASEP model

*The average TASEP motor velocity*

For the TASEP, gap sizes are geometrically distributed as (21)

$$P_{\mathrm{TASEP}}(g;a) = (1-a)\,a^{g}, \tag{1}$$

for some constant $a < 1$. Unless a motor is blocked by another motor ($g = 0$), it will hop forward with rate $k$, and the average velocity of motors can be calculated as

$$v^{\mathrm{b}} = k\sum_{g=1}^{\infty} P_{\mathrm{TASEP}}\,(g;a) = ka. \tag{2}$$

From this it follows that $a = v^{\mathrm{b}}/k$, and we can write

$$P_{\mathrm{TASEP}}(g; v^{\mathrm{b}}/k) = \left(1 - v^{\mathrm{b}}/k\right)\left(v^{\mathrm{b}}/k\right)^{g}. \tag{3}$$

*Intra- and trans-peloton gap sizes*

The inclusion of dynamic roadblocks will split the motor dynamics into an intra-peloton and a trans-peloton TASEP describing gap sizes below and above the roadblock shadow $\Delta$. In line with our heuristic argument, we assume there to be no roadblocks within a peloton. Apart from the leading motor, all motors within a peloton thus attempt to hop forward with rate $k_{\mathrm{ip}}$. The pelotons themselves are controlled by the leading motor, which faces a trans-peloton gap filled with roadblocks and thus attempts to hop forward with rate $k_{\mathrm{tp}}$. Assuming that the gap-size distribution is geometric both below and above the roadblock shadow, we can now write our normalized heuristic gap-size distribution for the intra- and trans-peloton regimes as

$$\begin{aligned} P_{\mathrm{ip}}(g; v^{\mathrm{b}}/k_{\mathrm{ip}}) &= \frac{\left(v^{\mathrm{b}}/k_{\mathrm{ip}}\right)^{g}}{\sum_{g=0}^{\Delta-1}\left(v^{\mathrm{b}}/k_{\mathrm{ip}}\right)^{g}} = \left(1 - v^{\mathrm{b}}/k_{\mathrm{ip}}\right)\left(v^{\mathrm{b}}/k_{\mathrm{ip}}\right)^{g} + O\left[\left(v^{\mathrm{b}}/k_{\mathrm{ip}}\right)^{\Delta}\right], \quad g < \Delta \\ P_{\mathrm{tp}}(g; v^{\mathrm{b}}/k_{\mathrm{tp}}) &= \frac{\left(v^{\mathrm{b}}/k_{\mathrm{tp}}\right)^{g}}{\sum_{g=\Delta}^{\infty}\left(v^{\mathrm{b}}/k_{\mathrm{tp}}\right)^{g}} = \left(1 - v^{\mathrm{b}}/k_{\mathrm{tp}}\right)\left(v^{\mathrm{b}}/k_{\mathrm{tp}}\right)^{g-\Delta}, \quad g \geq \Delta. \end{aligned} \tag{4}$$

The above equations are valid when $(k_{\rm ip}/k_{\rm tp})^{\Delta}$ is large, which we refer to as the stable peloton regime (SPR). The condition for the SPR can intuitively be seen as combining the strength of the attraction between motors and roadblocks ($k_{\rm ip}/k_{\rm tp}$) and its range ($\Delta$). Due to the SPR conditions exponential dependence on the roadblock shadow size, we expect physiological systems where the roadblock size is substantially larger than the motor step to always be in the SPR. In this limit, we have

$$\begin{aligned} P_{\rm ip}(g; v^{\rm b}/k_{\rm ip}) &= P_{\rm TASEP}(g; v^{\rm b}/k_{\rm ip}), \quad g < \Delta, \\ P_{\rm tp}(g; v^{\rm b}/k_{\rm tp}) &= P_{\rm TASEP}(g-\Delta; v^{\rm b}/k_{\rm tp}), \quad g \geq \Delta \end{aligned} \tag{5}$$

With these conditional distributions, we can now calculate the average gaps sizes for both regimes

$$\begin{aligned} \langle g \rangle^{\rm b}_{\rm ip} &= \sum_{\rm g=0}^{\infty} g P_{\rm TASEP}(g; v^{\rm b}/k_{\rm ip}) = \frac{v^{\rm b}}{k_{\rm ip} - v^{\rm b}}, \\ \langle g \rangle^{\rm b}_{\rm tp} &= \sum_{\rm g=\Delta}^{\infty} g P_{\rm TASEP}(g-\Delta; v^{\rm b}/k_{\rm tp}) = \Delta + \frac{v^{\rm b}}{k_{\rm tp} - v^{\rm b}}. \end{aligned} \tag{6}$$

Defining $p^{\rm b}$ as the probability of a gap in the bulk being a trans-peloton gap, we can write the average gap between motors as $\langle g \rangle^{\rm b} = (1-p^{\rm b})\langle g \rangle^{\rm b}_{\rm ip} + p^{\rm b}\langle g \rangle^{\rm b}_{\rm tp}$. Taking the average motor size into account, the average motor density is the inverse of the typical distance between the fronts of neighboring motors,

$$\rho^{\rm b}_{\rm m} = \frac{1}{\langle g \rangle^{\rm b} + \delta_{\rm m}} = \frac{1}{(1-p^{\rm b})\,\langle g \rangle^{\rm b}_{\rm ip} + p^{\rm b}\langle g \rangle^{\rm b}_{\rm tp} + \delta_{\rm m}}. \tag{7}$$

*The relative fraction of trans peloton gaps*

Combining the intra- and trans-peloton distributions we can write the complete gap-size distribution of the hierarchical TASEP as

$$P_{\rm hTASEP}(g; v^{\rm b}/k_{\rm ip}, v^{\rm b}/k_{\rm tp}, \Delta) = \begin{cases} (1-p^{\rm b}) P_{\rm TASEP}(g; v^{\rm b}/k_{\rm ip}), & g < \Delta \\ p^{\rm b} P_{\rm TASEP}(g-\Delta; v^{\rm b}/k_{\rm tp}), & g \geq \Delta \end{cases}. \tag{8}$$

In steady state, the probabilistic flow from intra- to trans-peloton gaps (a peloton is split in two) and from trans- to intra-peloton gaps (two pelotons merge) should balance. The motor ahead of a gap of size $\Delta - 1$ hops with an average rate $v$ and extends the gap to size $\Delta$, inducing the mean-field probabilistic flow $v^{\rm b} P_{\rm hTASEP}(\Delta-1; v^{\rm b}/k_{\rm ip}, v^{\rm b}/k_{\rm tp}, \Delta) = v^{\rm b}(1-p^{\rm b}) P_{\rm TASEP}(\Delta - 1; v^{\rm b}/k_{\rm ip})$. In turn, a motor behind a gap of size hops forward with an average rate and decreases the gap to size $\Delta - 1$, inducing the probabilistic flow $k_{\rm tp} P_{\rm hTASEP}(\Delta; v^{\rm b}/k_{\rm ip}, v^{\rm b}/k_{\rm tp}, \Delta) = k_{\rm tp} p^{\rm b} P_{\rm TASEP}(0; v^{\rm b}/k_{\rm tp})$. In the steady state these two flows should balance, and equating these rates gives

$$p^{\rm b} = \frac{[\langle g \rangle^{\rm b}_{\rm tp} - \Delta]}{[\langle g \rangle^{\rm b}_{\rm tp} - \Delta] + \langle g \rangle^{\rm b}_{\rm ip} \left(\frac{k_{\rm ip}}{v^{\rm b}}\right)^{\Delta}}. \tag{9}$$

Taken together, Equations 6, 7, and 9 relate the average density in the system to the velocity and the microscopic model parameters. In Figure 2 C in the main text we compare the gap-size distribution resulting from our heuristic arguments (solid lines) with ones generated through simulations of the BRM (dots) at different motor densities

## 2 Relation between heuristic arguments and mean-field solution of BRM

For periodic boundary conditions and irreversible roadblock binding, there exists a mean-field solution for the special case $\delta_{\rm m} = \delta_{\rm rb} = 1$ [2]. It is informative to consider our more general but less precise heuristic approach in the light of this mean-field solution. We first present a short summary of the mean-field solution, and then show how a few approximations give our heuristic results. The gap-size distribution for the BRM in the mean-field is given by

$$P_{\rm BRM}(g) = \frac{\prod_{y=1}^{g} \frac{v^{\rm b}}{k(y)}}{\sum_{g=0}^{\infty} \prod_{y=1}^{g} \frac{v^{\rm b}}{k(y)}}. \tag{10}$$

Here $k(g)$ is the average stepping rate of a motor into a gap of size $g$. This average stepping rate can be determined by calculating the probability of a roadblock with binding rate $k_{\rm b}$ being bound to a lattice site after a time $t$ since the last motor passed, $P_{\rm rb}(t) = 1 - e^{-k_{\rm b}t}$. If we make the mean-field assumption that the motor moves with the average velocity $v$ of the system, the probability of a roadblock being bound can be written in terms of the gap size to the motor ahead $P_{\rm rb}(g) = 1 - e^{-gk_{\rm b}/v^{\rm b}}$. The effective stepping rate of a motor as a function of the gap ahead is now given by

$$k(g) = P_{\rm rb}(g)k_{\rm tp} + (1 - P_{\rm rb}(g))\, k_{\rm ip} = k_{\rm tp} + (k_{\rm ip} - k_{\rm tp})\, e^{-gk_{\rm b}/v^{\rm b}} = k_{\rm tp} + (k_{\rm ip} - k_{\rm tp})\, e^{-g/(\Delta-1)} \tag{11}$$

where we in the last step introduced the roadblock shadow $\Delta = 1 + v^{\rm b}/k_{\rm b}$ for the BRM, and in direct analogy with our heuristic approach. If we now approximate $k(g)$ by a step function

$$k(g) = \begin{cases} k_{\rm ip}, & g < \Delta \\ k_{\rm tp}, & g \geq \Delta \end{cases} \tag{12}$$

the gap-size distribution is given by

$$P_{\rm BRM}(g) \propto \begin{cases} \left(v^{\rm b}/k_{\rm ip}\right)^{g}, & g < \Delta \\ \left(k_{\rm tp}/k_{\rm ip}\right)^{\Delta-1} \left(v^{\rm b}/k_{\rm tp}\right)^{g}, & g \geq \Delta \end{cases} \tag{13}$$

Enforcing normalization, Equation 13 implies Equation 4.

## 3 Observable bulk quantities

In addition to the motor density, there are other interesting observables that can be calculated if we know the average velocity in the bulk. Among them are the current of motors, $J = \rho_{\rm m}^{\rm b} v^{\rm b}$, and the average number of motors in a peloton, $\langle n_{\rm p}\rangle^{\rm b} = 1/p^{\rm b}$. Further, only gaps between pelotons are filled with roadblocks, and then typically only beyond the roadblock shadow. If we let $\rho_{\rm rb}^{\rm eq}$ be the equilibrium roadblock occupancy in the absence of motor activity, we can estimate the average roadblock occupancy as

$$\rho_{\rm rb}^{\rm b} = \rho_{\rm rb}^{\rm eq} \frac{p^{\rm b}[\langle g\rangle_{\rm tp}^{\rm b} - \Delta]}{\langle g\rangle^{\rm b} + \delta_{\rm m}}. \tag{14}$$

In supplementary Figure 1 we illustrate the relationships derived for various observables and check our arguments against simulations of the BRM. In Figure 1 A-D we show the effect of varying the trans-peloton hopping rates for long roadblock equilibration times. As predicted by

our analytical arguments, up to a critical motor density $\rho_1$ the velocity remains approximately constant (Figure 1 A), and the total current of motors grows linearly with the motor density (Figure 1 B), while the roadblock occupancy decreases linearly with motor density (Figure 1 C). At the critical density, and long before the track is completely covered by motors, all roadblocks are evicted. For motor densities above the critical density, the velocity and motor current follows the relationship for the TASEP without roadblocks (the ipTASEP) (Figure 1 A and B). In Figure 1 D we plot the typical peloton size up to the critical density, after which whole system acts as one large roadblock-excluding peloton. In Figure 1 E-H we vary the roadblock equilibration times. For rapid roadblock equilibration (red curves Figure 1 E-H) the roadblock shadow is small, gaps are largely filled with roadblocks, and the system is well described by a single TASEP with roadblocks in every gap (the tpTASEP) (dashed line in Figure 1 E). In this regime, the total density of roadblocks decreases weakly with motor density (red curve Figure 1 G), as roadblock shadows are small and the motor footprints are all that excludes the roadblocks. For intermediate roadblock equilibration times (the blue curves in Figure 1 E-H) the roadblock shadow is larger, resulting in peloton formation, a velocity that is less sensitive to motor density, and a system that is better at evicting roadblocks. The breakdown of our predictions for roadblock densities and peloton size in the case of fast roadblock binding (red and blue curves in Figure 1 G and H) is not surprising given that we here have small enough roadblock shadows to push the system outside the SPR.

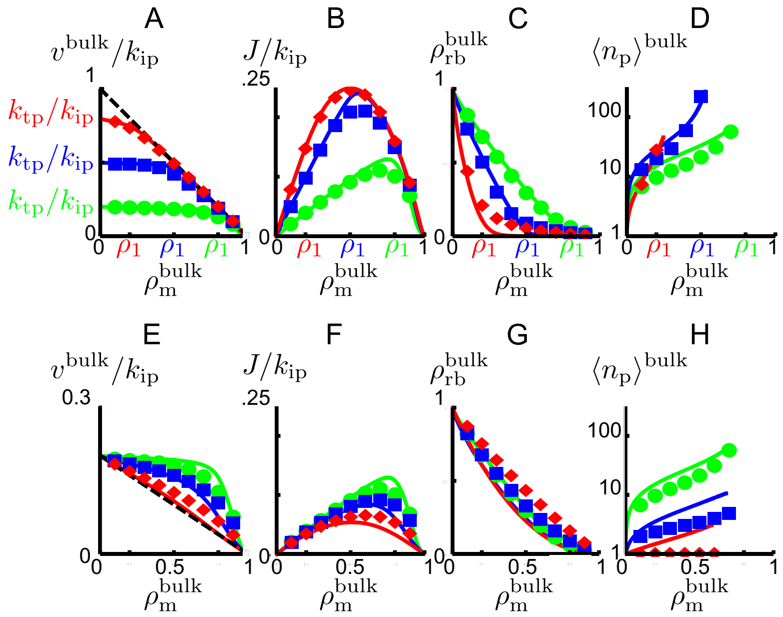


**Figure 1: General model captures bulk dynamics of the BRM.** Solid lines are analytical predictions and symbols are results from Monte Carlo simulations. Within the rows, each color represents the same parameter values, while green represents the same parameter values across all panels. **(A)-(D)** Systems in the SPR: the parameter values are $k_{\rm ip}\tau = 20$ for all curves, with $k_{\rm tp}/k_{\rm ip} = 0.8$ (red), $k_{\rm tp}/k_{\rm ip} = 0.5$ (blue), and $k_{\rm tp}/k_{\rm ip} = 0.2$ (green). The dashed line in (A) is the velocity relation for the ipTASEP. **(E)-(H)** Sweep from stable to non-stable pelotons: the parameter values are $k_{\rm tp}/k_{\rm ip} = 0.2$ for all curves, and $k_{\rm ip}\tau = 20$ (green), $k_{\rm ip}\tau = 10$ (blue), $k_{\rm ip}\tau = 2$ (red). The dashed line in (E) is the velocity relation for the tpTASEP. In (A) and (E) we show the velocity, in (B) and (F) the motor current, in (C) and (G) the roadblock occupancy and in (D) and (H) the peloton size, all as a function of motor density. In the SPR, it only makes sense to talk about pelotons when there are roadblocks in the system ($\rho_{\rm m}^{\rm bulk} < \rho_1$), and we only show the burst size as a function of motor activity for these densities. For small enough roadblock shadows (red and blue line in Figure (E)-(H) our description breaks down as the system falls outside the SPR.

# 4 Asymptotic behavior in the SPR

We here show how Equations 6, 7, and 9 can be solved explicitly in terms of the motor density rather than velocity in the limit SPR. For notational convenience we introduce the average excess trans-peloton gap $\langle\tilde{g}\rangle^{\rm b}_{\rm tp}$ and the critical density $\rho_0$, to write Equation (9) as

$$p^{\rm b} = \frac{\langle\tilde{g}\rangle^{\rm b}_{\rm tp}\rho_0}{1+\langle\tilde{g}\rangle^{\rm b}_{\rm tp}\rho_0}, \quad \langle\tilde{g}\rangle^{\rm b}_{\rm tp} = \langle g\rangle^{\rm b}_{\rm tp} - \Delta = \frac{v^{\rm b}}{k_{\rm tp}-v^{\rm b}}, \quad \rho_0 = \frac{1}{\langle g\rangle^{\rm b}_{\rm ip}}\left(\frac{v^{\rm b}}{k_{\rm ip}}\right)^{\Delta}. \tag{15}$$

In the SPR, the transition density $\rho_0$ is very small by definition. In the limit $\rho_0\langle\tilde{g}\rangle^{\rm b}_{\rm tp} \gg 1$, pelotons are small and $p^{\rm b}$ is close to one, and Equation 3 can be written as

$$1/\rho^{\rm b}_{\rm m} - \delta_{\rm m} \approx \langle g\rangle^{\rm b}_{\rm ip} \tag{16}$$

Here we have a system controlled by the tpTASEP. In the limit $\rho_0\langle\tilde{g}\rangle^{\rm b}_{\rm tp} \ll 1$, pelotons are large, $p^{\rm b}$ is small, and Equation 7 can be written as

$$1/\rho^{\rm b}_{\rm m} - \delta_{\rm m} \approx \langle g\rangle^{\rm b}_{\rm ip} + \langle\tilde{g}\rangle^{\rm b}_{\rm tp}(\langle\tilde{g}\rangle^{\rm b}_{\rm tp} + \Delta)\rho_0. \tag{17}$$

If the first two terms on the right hand side dominate, we have the standard ipTASEP. If the last term dominates, we have a composite system. Taken together, there are three limits given by

$$\begin{array}{ll} \langle g\rangle^{\rm b}_{\rm tp} \approx 1/\rho^{\rm b}_{\rm m} - \delta_{\rm m}, & \rho^{\rm b}_{\rm m} \ll \rho_0 \\ \langle\tilde{g}\rangle^{\rm b}_{\rm tp} \approx \frac{1}{\sqrt{\rho_0\rho^{\rm b}_{\rm m}}}, & \rho_0 \ll \rho^{\rm b}_{\rm m} \ll \rho_1 = \frac{1}{\langle g\rangle^{\rm b}_{\rm ip}+\delta_{\rm m}} \\ \langle g\rangle^{\rm b}_{\rm ip} \approx 1/\rho^{\rm b}_{\rm m} - \delta_{\rm m}, & \rho_1 \ll \rho^{\rm b}_{\rm m}. \end{array} \tag{18}$$

In the middle regime is large, and the average velocity (see Equation 6) is close to $k_{\rm tp}$,

$$v^{\rm b} = k_{\rm tp}\left(1 - \sqrt{\rho_0\rho^{\rm b}_{\rm m}}\right). \tag{19}$$

Using Equations 18 and 19, all observables can be written to leading order in $1/\rho_0$ as

$$\begin{array}{l} v^{\rm b} = k_{\rm tp}, \quad \langle g\rangle^{\rm b}_{\rm ip} = \dfrac{k_{\rm tp}}{k_{\rm ip}-k_{\rm tp}}, \quad \langle g\rangle^{\rm b}_{\rm tp} = \dfrac{1}{\sqrt{\rho_0\rho^{\rm b}_{\rm m}}}, \\ \langle n_{\rm p}\rangle^{\rm b} = \sqrt{\dfrac{\rho^{\rm b}_{\rm m}}{\rho_0}}, \quad \dfrac{\rho^{\rm b}_{\rm rb}}{\rho^{\rm eq}_{\rm rb}} = 1 - \dfrac{\rho^{\rm b}_{\rm m}}{\rho_1}, \quad \rho_0 = \dfrac{k_{\rm ip}-k_{\rm tp}}{k_{\rm tp}}\left(\dfrac{k_{\rm tp}}{k_{\rm ip}}\right)^{k_{\rm tp}\tau+\delta_{\rm rb}} \end{array} \tag{20}$$

In this regime, ($\rho_0 \ll \rho_{\rm m} \ll \rho_1$), the average bulk peloton size is large, and that is why we refer to this as the stable peloton regime.

# 5 The bulk state is never reached

With the parameter values in Table 1 in the main text, the bulk state is given by

$$\langle g\rangle^{\rm b}_{\rm ip} \approx 0.4, \quad \langle g\rangle^{\rm b}_{\rm tp} \approx 1\frac{5\cdot 10^{82}}{\sqrt{\rho_{\rm m}}} \gg 1, \quad v^{\rm b} \approx 3/{\rm s}, \quad \langle n_{\rm p}\rangle^{\rm b} \approx 5\cdot 10^{82}\sqrt{\rho_{\rm m}} \gg 1. \tag{21}$$

From the above it is clear that the average steady-state peloton size $\langle n_{\rm p}\rangle^{\rm b}$ is in general enormous throughout the experimentally accessible range, and that the true bulk-dynamics will never be reached over a finite gene. Judging by the size of bulk pelotons, polymerases that meet along the gene stay together until termination, invariably producing bursts of RNA production.

# 6 Initiation limited dynamics

Without roadblocks, and if the initiation rate $k_{\rm in}$ is limiting, the density and the distribution of motors at the start of the lattice are the same as in the bulk [3]. With the inclusion of roadblocks, the microscopic organization among motors and roadblocks can change from the start of the lattice to the bulk. Here we assume that the density of motors is low enough, such that once a motor is initiated it is only slowed down by other motors when it encounters a peloton. This condition means that once the initiation site is cleared, motors step away much faster at the beginning of the lattice than a new motor typically initiates. We will refer to this regime as the slow initiation regime, and we detail its extent below.

*The formation of pelotons*

A schematic kymograph for the TASEP with roadblocks and open boundary conditions is shown in supplementary Figure 2. At the start of the lattice, the time gaps between newly initiated motors are exponentially distributed with rate $k_{\rm in}$. As the motors move into the system, those motors that happened to have a roadblock bound ahead will start inducing peloton-forming traffic jams. For convenience we here call these jams proto-pelotons, and they will grow until all motors between roadblocks are absorbed into one peloton. Once all motors are collected into pelotons, we will refer to these as the fully formed pelotons. We here set out to determine the nature of this peloton formation, and what effects it has on both motor and roadblock density profiles.

In all the expressions below, the superscript 'in' refers to the first site after the initiation site for which $x = 0$. For a roadblock to bind to the first site after a motor just left, the motor first has to take $\delta_{\rm rb}$ steps and then a roadblock has to bind, all before another motor initiates. Considering the splitting probabilities for each step, we can write the probability of a roadblock binding between two motor initiation events as

$$p^{\rm in} = \left(\frac{1}{1 + k_{\rm in}\tau_{\rm m}^{\rm in}}\right)^{\delta_{\rm rb}} \frac{1}{1 + k_{\rm in}\tau}. \tag{22}$$

Here $\tau_{\rm m}^{\rm in}$ is the average time it takes a motor to take a step at the start of the track,

$$\tau_{\rm m}^{\rm in} = \frac{1 - p^{\rm in}}{k_{\rm ip}} + \frac{p^{\rm in}}{k_{\rm tp}}. \tag{23}$$

The definition of the slow initiation regime implies $\tau_{\rm m}^{\rm in} \ll 1/k_{\rm in}$, and Equation 22 can be simplified as

$$p^{\rm in} = \frac{e^{-\delta_{\rm rb}\tau_{\rm m}^{\rm in} k_{\rm in}}}{1 + k_{\rm in}\tau}. \tag{24}$$

Equation 23 and 24 can be used to solve for $p^{\rm in}$ explicitly in the steady state,

$$p^{\rm in} = \Lambda^{-1} W\left(\frac{k_{\rm in}\tau}{1 + k_{\rm in}\tau}\delta_{\rm rb}\,\Lambda\, e^{-k_{\rm in}\delta_{\rm rb}/k_{\rm ip}}\right), \quad \Lambda = \left(\frac{1}{k_{\rm tp}} - \frac{1}{k_{\rm ip}}\right)\frac{1}{\tau}, \tag{25}$$

where W is the Lambert W function. In the limit of low initiation rates we can write

$$p^{\rm in} = \frac{1}{1 + k_{\rm in}\tilde{\tau}}, \quad \tilde{\tau} = \tau + \delta_{\rm rb}/k_{\rm tp}. \tag{26}$$

Next we calculate the typical time it takes for $n_{\rm p}^{*}$ motors to aggregate into a peloton. Let $\Delta x$ be the typical distance that the proto-peloton back end moves between two motors joining (supplementary Figure 2). From the geometry of typical times and distances sketched in the kymograph of supplementary Figure 2, we can write

$$\left.\begin{array}{l}\Delta t\, k_{\mathrm{ip}} = \Delta x \\ \langle g\rangle_{\mathrm{ip}}^{\mathrm{in}} = \frac{k_{\mathrm{tp}}}{k_{\mathrm{ip}}-k_{\mathrm{tp}}} \\ (1/k_{\mathrm{in}} + \delta_{\mathrm{m}}/k_{\mathrm{ip}} + \Delta t)\, k_{\mathrm{tp}} = \Delta x + \delta_{\mathrm{m}} + \langle g\rangle_{\mathrm{ip}}^{\mathrm{in}}\end{array}\right\} \quad \Rightarrow \quad \Delta x = \frac{1}{k_{\mathrm{in}}\tau\,\Lambda} - \delta_{\mathrm{rb}}, \tag{27}$$

where $\Lambda$ is given in Equation (25). In the last step above, we used the fact that we are considering the slow initiation regime, $k_{\mathrm{in}} \ll k_{\mathrm{ip}}, k_{\mathrm{tp}}$. Proto-pelotons grow as more and more motor catch up, and again ignoring correlations, the final size $n_{\mathrm{p}}^*$ of a proto peloton is geometrically distributed as

$$P(n_{\mathrm{p}}^*) = p^{\mathrm{in}}(1-p^{\mathrm{in}})^{n_{\mathrm{p}^*}-1} \quad \Rightarrow \quad \langle n_{\mathrm{p}}^*\rangle = 1/p^{\mathrm{in}}. \tag{28}$$

Here the first factor in the probability function accounts for the probability of having a roadblock in a gap, and the following factors accounts for the probability of having no roadblocks in the preceding $n_{\mathrm{p}}^* - 1$ gaps. The probability that a proto-peloton is still growing at position $x$, or equivalently the probability that $n_{\mathrm{p}}^* > x/\Delta x$, is then given by

$$P_{\mathrm{grow}}(x) = \sum_{n_{\mathrm{p}^*} > x/\Delta x} p^{\mathrm{in}}(1-p^{\mathrm{in}})^{n_{\mathrm{p}}^*-1} = (1-p^{\mathrm{in}})^{x/\Delta x}. \tag{29}$$

Letting $\langle n_{\mathrm{p}}(x)\rangle$ be the size of the average proto-peloton at a distance from the initiation site, we can now write down the discrete evolution equation $\langle n_{\mathrm{p}}(x+\Delta x)\rangle = \langle n_{\mathrm{p}}(x)\rangle + 1\cdot P_{\mathrm{grow}}$, with $\langle n_{\mathrm{p}}(0)\rangle = 1$, giving

$$\langle n_{\mathrm{p}}(x)\rangle = \sum_{n=1}^{x/\Delta x} (1-p^{\mathrm{in}})^n + \langle n_{\mathrm{p}}(0)\rangle = \langle n_{\mathrm{p}}^*\rangle - \left[\langle n_{\mathrm{p}}^*\rangle - 1\right] e^{-x/x_{\mathrm{p}}}, \quad x_{\mathrm{p}} = -\Delta x/\ln\left(1-p^{\mathrm{in}}\right). \tag{30}$$

Though strictly only true for $x$ in multiples of $\Delta x$, we take the above equation to be valid for any position $x \geq 0$.

*The macroscopic effects of peloton formation*

We now turn to calculate how the gradual growth of the proto pelotons impacts the motor density and velocity. The motor number density $\rho_{\mathrm{m}}(x)$ at any position $x$ is defined as the fraction of time that a site is occupied by (say) the front of a motor. The proto-peloton size at position $x$ tells us that the fraction $\langle n_{\mathrm{p}}(x)\rangle p_{\mathrm{in}}$ motors take an average time $1/k_{\mathrm{tp}}$ to step, while the rest take $1/k_{\mathrm{ip}}$, giving the average stepping time

$$\tau_{\mathrm{m}}(x) = \frac{\langle n_{\mathrm{p}}(x)\rangle p^{\mathrm{in}}}{k_{\mathrm{tp}}} + \frac{1-\langle n_{\mathrm{p}}(x)\rangle p^{\mathrm{in}}}{k_{\mathrm{ip}}}, \quad \tau_{\mathrm{m}}(0) = \tau_{\mathrm{m}}^{\mathrm{in}}. \tag{31}$$

The total time $T$ between the seeding of two pelotons (supplementary Figure 2) averages over peloton sizes to

$$\langle T\rangle = \langle n_{\mathrm{p}}^*\rangle \left(1/k_{\mathrm{in}} + \delta_{\mathrm{m}}\tau_{\mathrm{m}}^{\mathrm{in}}\right). \tag{32}$$

The fraction of time that the track is occupied by motors at position $x$ is then

$$\rho_{\mathrm{m}} = \frac{\langle n_{\mathrm{p}}\rangle \tau_{\mathrm{m}}(x)}{\langle T\rangle} = \frac{\tau_{\mathrm{m}}(x)}{1/k_{\mathrm{in}} + \delta_{\mathrm{m}}\tau_{\mathrm{m}}^0} = \rho_{\mathrm{m}}^* + (\rho_{\mathrm{m}}^{\mathrm{in}} - \rho_{\mathrm{m}}^*)e^{-x/x_{\mathrm{p}}} \tag{33}$$

where the $\rho_{\mathrm{m}}^*$ denotes the motor density where the proto pelotons have fully formed. The average motor hopping rate can similarly be written as

$$k_\mathrm{m}(x) = p^\mathrm{in}\langle n_\mathrm{p}(x)\rangle k_\mathrm{tp} + (1 - p^\mathrm{in}\langle n_\mathrm{p}(x)\rangle)k_\mathrm{ip} = k^* + (k^\mathrm{in} - k^*)e^{-x/x_\mathrm{P}}. \tag{34}$$

Though true for the TASEP, note that here the average motor hopping rate (Equation 34) is typically not the same as the average velocity $v(x) = \tau_\mathrm{m}^{-1}(x)$ in our system. With the velocity defined this way, it satisfies the standard relation $J = \rho_\mathrm{m}(x)v(x)$ where $J = \langle n_\mathrm{p}\rangle^*/\langle \mathrm{T}\rangle$ is the flux of motors.

In supplementary Figure 2 we indicate the times and positions where there typically are roadblocks in pink. Moving away from the initiation site, the fraction of time a site is occupied by roadblocks grows because motors that have not yet caught up with a proto peloton move faster than the proto pelotons themselves. The moving front of equilibrating roadblocks (intersection of white and pink region in supplementary Figure 2) is typically offset with respect to the last motor of the peloton by a distance $\delta_\mathrm{rb}$ (see dashed square in supplementary Figure 2). Taking the offset $\delta_\mathrm{rb}$ into account, Equation 29 implies that a fraction$P_\mathrm{grow}(x+\delta_\mathrm{rb})$ of the proto pelotons is still evolving when the front of equilibrating roadblocks is at position $x$. The increase of the average time a site at position $x$ (see supplementary Figure 2) is occupied by a roadblock then grows with distance as

$$t_\mathrm{rb}(x + \Delta x) = t_\mathrm{rb}(x) + (1/k_\mathrm{tp} - 1/k_\mathrm{ip})\, \Delta x P_\mathrm{grow}(x + \delta_\mathrm{rb}), \quad t_\mathrm{rb}(0) = 1/k_\mathrm{in}. \tag{35}$$

This expression can be summed in the same manner as we previously summed to solve for $\langle n_\mathrm{p}(x)\rangle$ in Equation 30, yielding

$$t_\mathrm{rb}(x) = [1/k_\mathrm{tp} - 1/k_\mathrm{ip}]\, \Delta x e^{-\delta_\mathrm{rb}/x_\mathrm{P}} \left(\langle n_\mathrm{p}^*\rangle - 1\right)\left(1 - e^{-x/x_\mathrm{P}}\right) + 1/k_\mathrm{in}. \tag{36}$$

The total fraction of time a site is covered by roadblocks is now

$$\rho_\mathrm{rb}(x) = \rho_\mathrm{rb}^\mathrm{eq}\frac{t_\mathrm{rb}(x)}{\langle T\rangle} = \rho_\mathrm{rb}^* - (\rho_\mathrm{rb}^\mathrm{in} - \rho_\mathrm{rb}^*)e^{-x/x_\mathrm{P}}. \tag{37}$$

Relative changes are often easier to measure experimentally than absolute changes, therefore we here also give the relative changes in velocity and density at the beginning, and compared to well after the pelotons are formed

$$\begin{aligned}
\frac{\rho_\mathrm{m}^* - \rho_\mathrm{m}^\mathrm{in}}{\rho_\mathrm{m}^*} &= (1 - p^\mathrm{in})\left(1 - \frac{k_\mathrm{tp}}{k_\mathrm{ip}}\right), \quad \frac{v^* - v^\mathrm{in}}{v^*} = -(1 - p^\mathrm{in})\left(\frac{k_\mathrm{ip}}{k_\mathrm{tp}} - 1\right), \\
\frac{\rho_\mathrm{rb}^* - \rho_\mathrm{rb}^\mathrm{in}}{\rho_\mathrm{rb}^*} &= \frac{1}{1 + \frac{p^\mathrm{in}}{1-p^\mathrm{in}}\frac{\Delta x + \delta_\mathrm{m}}{\Delta x} \cdot e^{\delta_\mathrm{rb}/x_\mathrm{P}}}.
\end{aligned} \tag{38}$$

To have an appreciable motor density and velocity evolution, we need only the typical peloton to have a size of a few motors. In the limit of low initiation rates, the above can be written as

$$\begin{aligned}
\frac{\rho_\mathrm{m}^* - \rho_\mathrm{m}^\mathrm{in}}{\rho_\mathrm{m}^*} &= \frac{k_\mathrm{in}\tilde{\tau}}{1 + k_\mathrm{in}\tilde{\tau}}\left(1 - \frac{k_\mathrm{tp}}{k_\mathrm{ip}}\right), \quad \frac{v^* - v^\mathrm{in}}{v^*} = -\frac{k_\mathrm{in}\tilde{\tau}}{1 + k_\mathrm{in}\tilde{\tau}}\left(\frac{k_\mathrm{ip}}{k_\mathrm{tp}} - 1\right), \\
\frac{\rho_\mathrm{rb}^* - \rho_\mathrm{rb}^\mathrm{in}}{\rho_\mathrm{rb}^*} &= \frac{k_\mathrm{in}\tilde{\tau}}{e^{\delta_\mathrm{rb}/x_\mathrm{P}} + k_\mathrm{in}\tilde{\tau}}.
\end{aligned} \tag{39}$$

We see that relative changes along the track grow in magnitude with the initiation rate, but that the effect saturates around $k_\mathrm{in} \approx 1/\tilde{\tau}$ for motor-density and velocity changes, while the roadblock density saturates later, around $k_\mathrm{in} \approx e^{\delta_\mathrm{rb}/x_\mathrm{P}}/\tilde{\tau}$. Interestingly, we see that the total shift in motor density and velocity along the track is set solely by the ratio of motor stepping rates with and without roadblocks ahead.

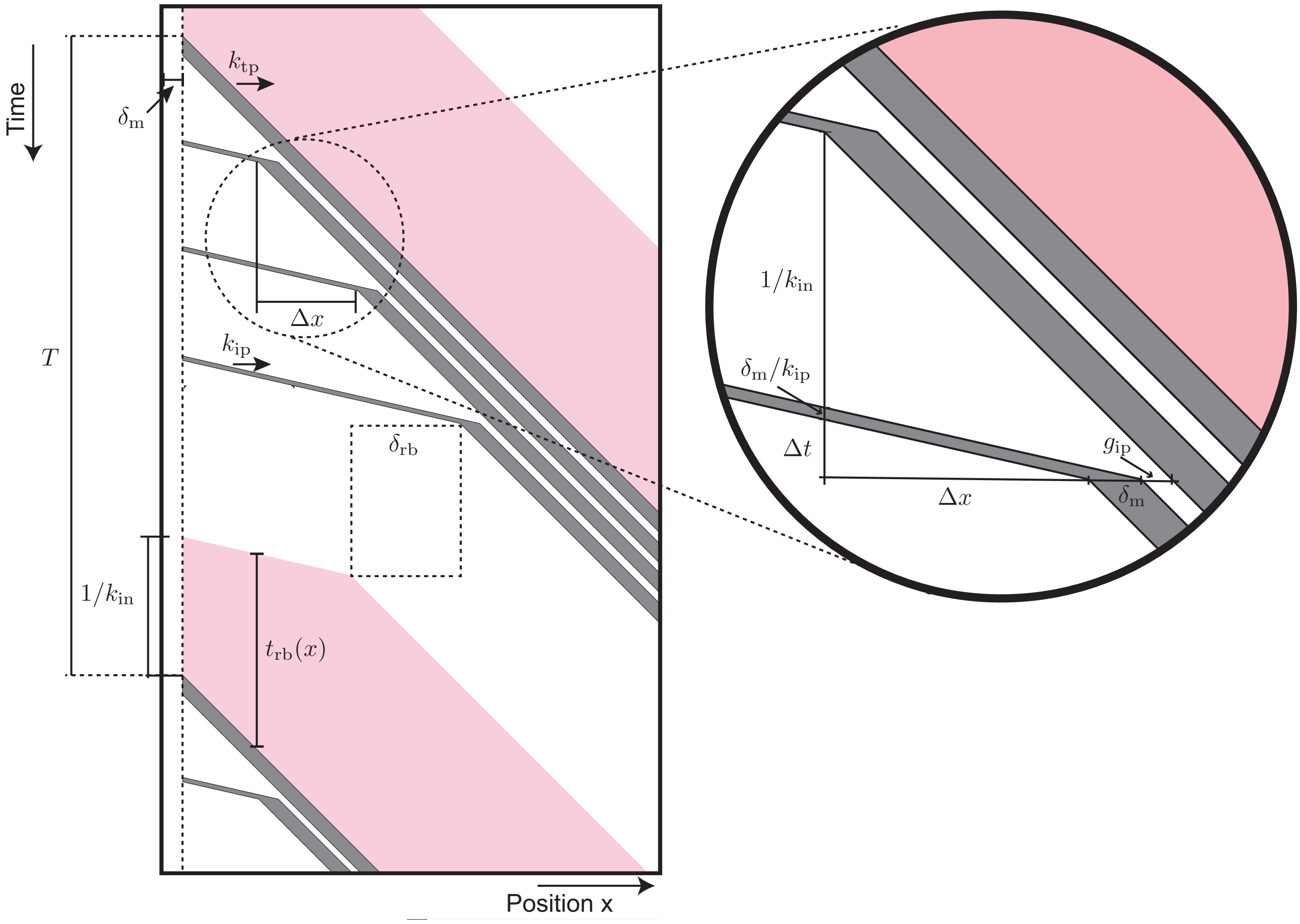


**Figure 2: A schematic kymograph of the system with open boundary conditions.** Motors (grey) initiate on the left side of the lattice, with an initiation site free of roadblocks. As motors travel into the system, their speed depends on if there is a roadblock (pink) ahead of them. With a roadblock ahead, the motor speed is $k_{\rm tp}$, while without roadblock ahead it is $k_{\rm ip}$. The typical time that a roadblock at position $x$ is bound before being evicted by the next peloton is given by $t_{\rm rb}(x)$. The right figure is a magnification of the region defining $\Delta x$, which is the typical distance a motor travels to catch up with the proto peloton since the last motor caught up.

# 7 Bursts from terminating pelotons

The peloton-forming dynamics of our model will manifest as burst of motor activity if viewed from a specific position (for example the transcription-termination site). Using the average velocity of the system, we can translate the average gap-sizes to average time gaps between motors arrivals

$$\tau_{\rm ip} = \frac{\langle g \rangle_{\rm ip} + \delta_{\rm m}}{v}, \quad \tau_{\rm tp} = \frac{\langle g \rangle_{\rm tp} + \delta_{\rm m}}{v}. \tag{40}$$

Letting $k_{\rm tr}$ be the rate of reaction when the process is in the on state, $k_{\rm off}$ be the rate at which the system transitions to the off state, and $k_{\rm on}$ be the rate at which the system transitions back to the on state, we can relate our first-principles model to the phenomenological two-state model traditionally used to describe transcriptional bursts (Figure 4 C in the main text) [4]. As both models generate double exponentials, we relate them to each other by equating the time constant and the corresponding relative probabilistic weight. Since we are interested in describing transcriptional bursts, we here consider the limit where the two-state model gives clearly separated bursts, $k_{\rm on} \ll k_{\rm off} + k_{\rm tr}$. In this limit we have, to leading order in $k_{\rm on}$,

$$p=\frac{k_{\rm off}}{k_{\rm off}+k_{\rm tr}},\quad \tau_{\rm ip}=\frac{1}{k_{\rm off}+k_{\rm tr}},\quad \text{and}\quad \tau_{\rm tp}=\frac{1}{k_{\rm on}}\left(1+\frac{k_{\rm off}}{k_{\rm tr}}\right). \tag{41}$$

The relations in Equation 41 can be inverted to give

$$k_{\rm tr}=\frac{1-p}{\tau_{\rm ip}},\quad k_{\rm off}=\frac{p}{\tau_{\rm ip}},\quad \text{and}\quad k_{\rm on}=\frac{1}{(1-p)\tau_{\rm tp}}. \tag{42}$$

For the bulk we can in principle calculate the corresponding two-state model using Equation 6, though this state is likely never reached. The physiologically more interesting situation is just after the initial pelotons have fully formed. The relative probability of a gap being between pelotons is then (see Equation 26)

$$p=p^{\rm in}=\frac{1}{1+k_{\rm in}\tilde{\tau}}. \tag{43}$$

Once the initial pelotons have formed beyond $x_{\rm p}$, we know that the average velocity is $k_{\rm tp}$, and we have

$$\langle g\rangle^*_{\rm ip}=\frac{k_{\rm tp}}{k_{\rm ip}-k_{\rm tp}}\quad\Rightarrow\quad \tau^*_{\rm ip}=\frac{1}{k_{\rm ip}-k_{\rm tp}}+\frac{\delta_{\rm m}}{k_{\rm tp}}. \tag{44}$$

The trans-peloton gaps can be written as (see supplementary Figure 2)

$$\begin{aligned} \langle T\rangle &= \tau^*_{\rm tp}+\tau^*_{\rm ip}\left(\langle n_{\rm p}\rangle^*-1\right)\\ \Rightarrow\quad \tau^*_{\rm tp}=\langle T\rangle-\tau^*_{\rm ip}k_{\rm in}\tilde{\tau} &= \left(\frac{1}{k_{\rm in}}+\frac{\delta_{\rm m}}{k_{\rm tp}}\right)+\left(\frac{1}{k_{\rm in}}-\frac{1}{k_{\rm ip}-k_{\rm tp}}\right)k_{\rm in}\tilde{\tau} \end{aligned} \tag{45}$$

Combining Equations 42-45 we have

$$k_{\rm tr}=\frac{k_{\rm in}\tilde{\tau}}{1+k_{\rm in}\tilde{\tau}}\frac{1}{\tau^*_{\rm ip}},\quad k_{\rm off}=\frac{1}{1+k_{\rm in}\tilde{\tau}}\frac{1}{\tau^*_{\rm ip}},\quad \text{and}\quad k_{\rm on}=\frac{1+k_{\rm in}\tilde{\tau}}{k_{\rm in}\tilde{\tau}}\frac{1}{\tau^*_{\rm tp}}. \tag{46}$$

In the main text we are interested in the case where motors are large and the initiation rate is low compared to motor stepping rates. In this limit we have

$$k_{\rm tr}=\frac{k_{\rm in}\tilde{\tau}}{1+k_{\rm in}\tilde{\tau}}\frac{k_{\rm tp}}{\delta_{\rm m}},\quad k_{\rm off}=\frac{1}{1+k_{\rm in}\tilde{\tau}}\frac{k_{\rm tp}}{\delta_{\rm m}},\quad \text{and}\quad k_{\rm on}=\frac{1}{\tilde{\tau}+\frac{k_{\rm in}\tilde{\tau}}{1+k_{\rm in}\tilde{\tau}}\frac{\delta_{\rm m}}{k_{\rm tp}}}\approx\frac{1}{\tilde{\tau}}. \tag{47}$$

Here the approximate relation should be valid in the case of transcription through nucleosomes as here the roadblock are substantially larger than the motors, and $\tilde{\tau}$ is consequently substantially larger than $\delta_{\rm m}/k_{\rm tp}$.

# 8 Monte Carlo Simulations

We validate our heuristic arguments using a random-sequential-update Monte Carlo scheme with fixed time step $dt$ to simulate our model. During a Monte Carlo step on a lattice of size $L+1$ there are $L+1$ possible events: all the motors on the lattice can make a step forward, bound roadblocks can unbind, roadblocks in solution can bind to an empty lattice site, and a motor can bind at the start of the lattice. The time step is chosen small enough that the probability of any event occurring with rate $k$ in a time $dt$ can be approximated as $kdt$. In our simulation $k_{\rm ip}$ is the fastest rate, and we choose $k_{\rm ip}dt=0.1$. We verified that our results are robust towards changes in $dt$. Without roadblocks, the time to equilibration for periodic systems scales with the system size as $L^{3/2}$ [1]. With roadblocks, the time to equilibration is expected to be larger due to the slow peloton dynamics. For the simulations with periodic boundary conditions we waited $L^2/dt$ iterations for the system to reach steady state and let simulations run a total amount

of $10L^2/dt$ iterations, and checked that the peloton size did not change for longer equilibration times. The velocities presented in supplementary Figure 1 and Figure 5 in the main text are calculated by averaging over the instantaneous hopping rates of the motors.

## References


[1] R A Blythe and M R Evans. Nonequilibrium steady states of matrix-product form: a solver's guide. *Journal of Physics A: Mathematical and Theoretical*, 40(46):R333–R441, nov 2007.

[2] O J O Loan, M R Evans, and M E Cates. Jamming transition in a homogeneous one-dimensional system : The bus route model. *Physical Review E*, 58(2):1404–1418, 1998.

[3] C T MacDonald, J H Gibbs, and A C Pipkin. Kinetics of Biopolymerization on Nucleic Acid Templates. *Biopolymers*, 6:1–25, 1968.

[4] Brian Munsky, Gregor Neuert, and Alexander van Oudenaarden. Using gene expression noise to understand gene regulation. *Science*, 336(6078):183–7, April 2012.
